# *In* *vitro* biocompatibility evaluation of a heat‐resistant 3D printing material for use in customized cell culture devices

**DOI:** 10.1002/elsc.202100104

**Published:** 2022-03-31

**Authors:** Steffen Winkler, Katharina V. Meyer, Christopher Heuer, Carlotta Kortmann, Michaela Dehne, Janina Bahnemann

**Affiliations:** ^1^ Institute of Technical Chemistry Leibniz University Hannover Hannover Germany; ^2^ Cell Culture Technology Faculty of Technology Bielefeld University Bielefeld Germany

**Keywords:** 3D printing, biocompatibility, cell culture, heat steam sterilization, rapid prototyping

## Abstract

Additive manufacturing (3D printing) enables the fabrication of highly customized and complex devices and is therefore increasingly used in the field of life sciences and biotechnology. However, the application of 3D‐printed parts in these fields requires not only their biocompatibility but also their sterility. The most common method for sterilizing 3D‐printed parts is heat steam sterilization—but most commercially available 3D printing materials cannot withstand high temperatures. In this study, a novel heat‐resistant polyacrylate material for high‐resolution 3D Multijet printing was evaluated for the first time for its resistance to heat steam sterilization and in vitro biocompatibility with mouse fibroblasts (L929), human embryonic kidney cells (HEK 293E), and yeast (*Saccharomyces cerevisiae* (*S. cerevisiae*)). Analysis of the growth and viability of L929 cells and the growth of *S. cerevisiae* confirmed that the extraction media obtained from 3D‐printed parts had no negative effect on the aforementioned cell types, while, in contrast, viability and growth of HEK 293E cells were affected. No different effects of the material on the cells were found when comparing heat steam sterilization and disinfection with ethanol (70%, v/v). In principle, the investigated material shows great potential for high‐resolution 3D printing of novel cell culture systems that are highly complex in design, customized and easily sterilizable—however, the biocompatibility of the material for other cell types needs to be re‐evaluated.

**Abbreviations**
EM: extraction medium
S. cerevisiae: Saccharomyces cerevisiae

## 1 INTRODUCTION

Additive manufacturing or 3D printing is considered a revolutionizing technology already changing the way of fabrication in diverse industrial fields. It enables the bottom‐up single‐step production of most complex building parts, creating structure elements, such as inaccessible cavities, where standard top‐down fabrication technologies would fail. In addition, product development processes benefit from rapid prototyping due to the high degree of customizability of the design. There are multiple different additive manufacturing technologies, such as Fused Deposition Modeling (FDM), Stereolithography (SLA), MultiJet Printing (MJP), Two‐Photon Polymerization, and many more [1], some of them with printing resolutions down to the micro‐ to nanometer scale [2, 3, 4]. Furthermore, diverse materials (e.g., polymers [5], silicones [6], ceramics [7], or even metals [8]) can be printed, enabling products that are translucent, flexible, highly stable, or conductive. In addition, multi‐material printing enables printing different materials at the same time, which allows gaskets or conductive paths to be integrated directly into a device [9].

3D printing is increasingly used for medical applications, such as dental prosthetics [10] or transplants for surgery [11, 12]. It is also used in life sciences and biotechnology, for example, to develop 3D‐printed bioreactors [13] and "lab‐on‐a‐chip" or "organ‐on‐a‐chip" systems for cell cultivation [14, 15, 16] and tissue engineering [17]. To enable microscopic or spectroscopic analysis, transparent materials are preferred for use in cell culture applications. Therefore, 3D‐ printed cell culture devices are predominantly printed from transparent photopolymers [13, 18, 19].

Especially for applications in cell culture and medical technology, the sterility of a product must be guaranteed. Typically, heat steam sterilization (autoclaving) is used to sterilize bioreactors and equipment because it is easily accessible and applicable. However, this method has significant disadvantages, such as deformation or degradation of many polymers under heat or humidity [20, 21] Therefore, sterilization or disinfection of 3D‐printed objects is often achieved by UV irradiation or chemicals (e.g., ethanol or ethylene dioxide) [22]. Despite the challenges in developing a heat‐resistant 3D printable polymer material, several heat‐resistant materials for additive manufacturing have already been reported, such as polyetheretherketones(PEEK), fluorinated polymers, polyurethanes, and polyacrylates [23, 24, 25, 26, 27, 28, 29]. Another important requirement for the use of a material for cell culture or medical device applications is its biocompatibility.

**PRACTICAL APPLICATION**

3D printing enables rapid manufacturing of highly individual and complex designs and is therefore revolutionizing the production and prototyping of customized parts for various applications in different disciplines. In the medical and biotechnology sectors, where 3D printing can be used to fabricate cell culture devices or bioreactors, the availability of sterile products is of particular importance. Heat steam sterilization is the easiest way to sterilize 3D‐printed parts. However, among the wide variety of 3D printing materials, few are commercially available that can withstand heat steam sterilization and have shown biocompatibility. In this study, a polyacrylate material (VisiJet M2S‐HT90) shows great potential to fulfill both criteria for cell culture applications with adherent fibroblast cells and yeast cells, and is therefore a promising material for the development of customized cell culture devices.

A generally approved definition of the concept of biocompatibility was adopted in 1986 at a consensus conference on “Definitions in Biomaterials” organized by D. F. Williams: “Biocompatibility is the ability of a material to perform with an appropriate host response in a specific application” [30, 31]. This definition already implies that biocompatibility is a characteristic of a system and not of a material per se. Additionally, the response of an organism to a 3D printing material is particularly dependent on the duration and nature of the interaction and therefore needs to be uniquely defined for each application and product [31, 32, 33]. To develop suitable assays and to gather information on biocompatibility test methods, the extensive information provided by international standards—such as ISO 10993—can be consulted [34].

In most cases, initial cytotoxicity screening is based on cell culture methods because these methods are sensitive, reliable and reproducible [35]. Continuous (immortalized) cell lines such as HeLa, L929, 3T3, WI‐38 or Chinese hamster ovary (CHO) cells are usually selected for these screening steps [35]. For further investigations, cells are selected depending on the anticipated use of the material under study. For example, fibroblasts such as mouse L929 cells are an appropriate choice for skin contact materials because they play a physiological role in the wound healing process around implanted devices [35]. One reason for a cytotoxic effect of a material may be the formation of substances that are leachable or extractable from the material. Common leachables and extractables that may originate from polymers include additives, processing aids, and to a lesser extent monomers and oligomers [35].

A variety of in vitro methods are available for testing cytotoxic effects, ranging from counting viable/dead cells under the microscope to biochemical assays, flow cytometric analysis, and real‐time live‐cell imaging technology [33, 35–37]. While microscopic observations – including counting of viable/dead cells using vital dyes such as trypan blue – and observations of changes in cell morphology provide an initial assessment of cytotoxic effects, biochemical assays provide more reliable and specific information [33, 35, 36]. Flow cytometric analysis and real‐time live‐cell imaging technologies offer even more specific data, but typically require costly instrumentation and more manual labor compared to traditional screening assays.

Despite the immense number of 3D printing materials already available, materials that are biocompatible and can also be used in heat steam sterilization procedures are hard to find. The aim of this study is to investigate the potential of a novel, heat‐resistant polyacrylate material (VisiJet M2S‐HT90) for cell culture applications. For this purpose, the material was printed using a high‐resolution MultiJet 3D printer and then post‐processed to remove the support material (VisiJet M2 Sup). After this post‐processing procedure, the 3D‐printed parts were sterilized by heat steam sterilization or disinfected with ethanol (70% v/v) to reveal any effect of the disinfection/sterilization process on the 3D printing material. Subsequently, all of the post‐processed objects were analyzed, and extraction media were obtained according to the ISO 10993:12 standards. The suitability of the material for cell culture applications with *Saccharomyces cerevisiae*(*S. cerevisiae*), suspension human embryonic kidney (HEK) cells and mouse L929 cells was then investigated.

## 2 MATERIALS AND METHODS

### 2.1 Experimental procedure

**FIGURE 1:**
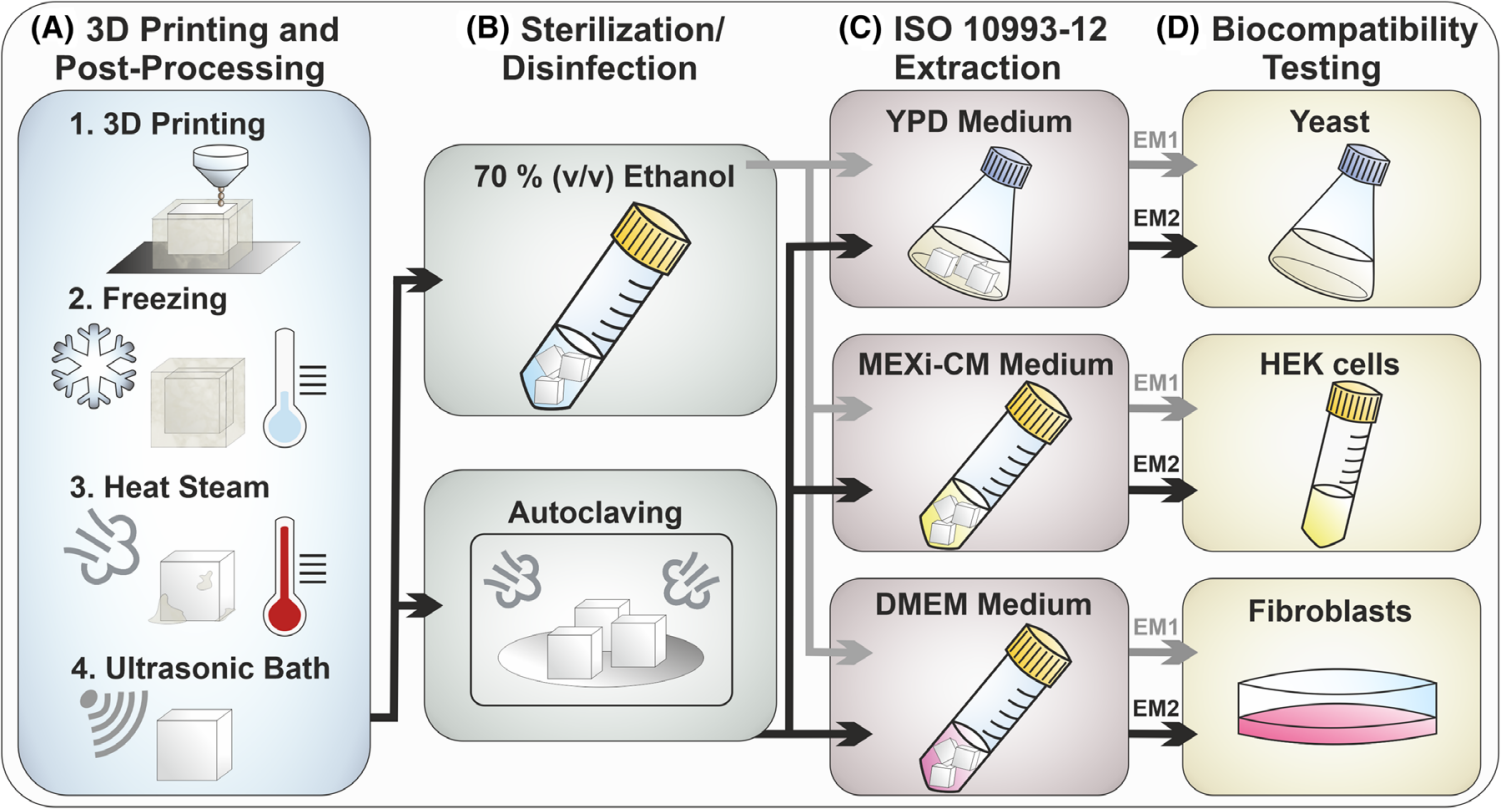
Schematic representation of the experimental procedure. The whole procedure includes 3D printing, post‐processing and sterilization/disinfection of the cubes (Section 2.2), preparation of extraction media in accordance to ISO 10993‐12 (Section 2.3) and biocompatibility testing using three different cell types (Section 2.5 ‐ 2.7). (EM 1: Extraction medium obtained by incubation of ethanol (70 %, v/v) disinfected 3D‐printed cubes; EM 2: Extraction medium obtained by incubation of autoclaved 3D‐printed cubes)

The experimental procedure is illustrated in Figure 1. The experiments started with 3D printing of 5 x 5 x 5 mm cubes (representing a total surface area of 1.5 cm^2^ per cube) with translucent polyacrylate material and support material. Subsequently, the printed cubes were cleaned in a post‐processing process and sterilized or disinfected either by autoclaving (30 min, 121°C) or incubation in ethanol (70% v/v; Merck KGaA, Darmstadt, Germany). To study the in vitro biocompatibility of the 3D printing material, extraction media were prepared according to ISO 10993:12. These were used to evaluate the effect of the 3D printing material on adherent mouse fibroblast cells (L929), suspension human embryonic kidney cells (HEK 293E), and suspension yeast cells (*S. cerevisiae* NCYC 1024).

### 2.2 3D printing, post‐processing and sterilization/disinfection of 3D‐printed objects

Cubes with 5 x 5 x 5 mm were designed using SolidWorks 2020 (Dassault Systemes Deutschland GmbH, Stuttgart, Germany) and 3D‐printed using the high‐resolution MultiJet 3D printer ProJet® MJP 2500 Plus (3D Systems, Rock Hill, SC, USA). The resolution of the printer in xyz is 800 x 900 x 790 DPI creating layers of 32 μm [38]. The 3D printing material tested within this study is referred to by the manufacturer as VisiJet® M2S‐HT90. According to the safety data sheet, the non‐polymerized model material contains several hazardous chemicals [39]. However, in the printed form, the model material is declared as biocompatible in accordance with USP‐class IV by the manufacturer [40]. In addition, the material is declared as heat‐stable with a heat distortion stability of 0.45 MPa at 90‐100°C [41]. As support material, VisiJet® M2 Sup was used, which according to the safety data sheet is a hydroxylated resin that has no evidenced toxic effects [42].

After the printing process was completed, the printing plate was incubated for 10 min at ‐18°C. This allows to remove the cubes from the plate and transfer them in a heat steam bath of an EasyClean unit (3D systems, Rock Hill, SC, USA) for 45 min.

Subsequently, the objects were incubated in an ultrasonic bath (Bandelin electronic, Berlin, Germany) with detergent (1% (v/v); Fairy Ultra Plus, Procter and Gamble, CT, USA) for 30 min at 65°C. Afterwards, water (Arium Sartorius Stedim Biotech GmbH, Göttingen, Germany) and detergent were renewed, and the incubation step was repeated. Following incubation with detergent, the 3D‐printed objects were incubated in water for 30 min at 65°C and then dried for 30 min at 70°C.

In this study, chemical disinfection by incubation in ethanol (70 %, v/v) for 1 h at room temperature and sterilization by autoclaving the objects for 30 min at 121°C (Systec VX‐150, Systec GmbH, Linden, Germany) were compared. The material showed no distortion after heat steam sterilization. Finally, all cubes were washed thoroughly with sterile phosphate‐buffered saline (PBS; Life Technologies Limited, Paisley, United Kingdom).

### 2.3 Preparation of extraction media (EM) for biocompatibility studies

To study potential leaching properties of the 3D printing material, extraction medium (EM) was prepared according to ISO 10993‐12:2021(E) (Biological evaluation of medical devices — Part 12: Sample preparation and reference materials) [34]. Following the post‐processing and sterilization/disinfection steps, extraction media were obtained by incubating the 3D‐printed cubes for 72 ± 2 h at 37°C (with a surface area/volume ratio of 3 cm^2^ ml^−1^) in the respective culture media. EM obtained by incubation of ethanol disinfected 3D‐printed cubes is referred to as EM 1. EM obtained by incubation of autoclaved 3D‐printed cubes is referred to as EM 2. In all biocompatibility experiments, the respective cell culture medium incubated for 72 h at 37°C without 3D‐printed cubes served as a control.

### 2.4 Flow cytometric analysis of extraction media

To detect particles that may have detached from the 3D printing material during incubation in the media, the EM (for culturing L929 and HEK 293E) and the corresponding control media were analyzed using a BD Accuri™ C6 (Becton Dickinson, NY, USA) flow cytometer. All samples were filtered through a 70 μm cell strainer (Corning Incorporated, Corning, USA) prior to the experiment, and 20 μL of each medium was analyzed. Each particle within the sample was detected by the instrument as an event. BD Accuri C6 software (Becton Dickinson, USA) was used for data analysis, and all media samples were compared to a size calibration sample containing polystyrene microspheres of known diameter (1.0, 2.0, 4.0, 6,0, 10, and 15 μm (Thermo Fisher Scientific Inc., Waltham, USA)).

### 2.5 L929 culture conditions and viability assessment

#### 2.5.1 Cell line and cell culture conditions

L929 cells (DSMZ‐German Collection of Microorganisms and Cell Cultures GmbH, Braunschweig, Germany, No. ACC2) were routinely cultivated in 75 cm^2^ cell culture flasks (Corning, CellBind Surface, Corning, NY, USA) in Dulbecco's Modified Eagle's Medium (DMEM; Sigma‐AldrichChemie GmbH, Steinheim, Germany), supplemented with 10% fetal calf serum (Sigma‐AldrichChemie GmbH, Steinheim, Germany) and 1% Penicillin/Streptomycin (Sigma‐AldrichChemie GmbH, Steinheim, Germany) in a 5% CO_2_, humidified atmosphere at 37°C (Heracell 240 incubator, Thermo Fisher Scientific Inc., Waltham, USA) and harvested at 70‐85 % confluency by Trypsin/EDTA solution (Biochrom GmbH, Berlin, Germany) treatment. Experiments were performed with cells of passage numbers below 13. 24 h prior to the start of an experiment, cells were seeded in 96‐well plates (Sarstedt AG and Co. KG, Nümbrecht, Germany) at a density of 15,000 cells per well and 7,500 cells per well in 200 μL cell culture medium.

#### 2.5.2 CellTiter blue (CTB) viability assay

Cell viability of the L929 cells was determined by CellTiter‐Blue® cell viability assay (Promega GmbH, Mannheim, Germany) using the background and standard controls given in the accompanying manual. In metabolically active cells, blue resazurin is reduced to purple fluorescent resorufin [33, 43, 44]. The resulting fluorescence intensity is an indicator of the number of viable cells. The formation of resorufin was monitored using a fluorescence plate reader (Fluoroskan Acent, Thermo Fisher Scientific Inc., Waltham, MA, USA) at an extinction wavelength of 544 nm and an emission wavelength of 590 nm.

The L929 cells were cultured in the related EM (see Section 2.3) or control medium for 24 h (15,000 cells per well) or 48 h (7,500 cells per well), afterwards all medium was removed, 100 μL of fresh DMEM containing 10 % CTB stock solution was added to each well and the cells were incubated for 1 h before fluorescence was measured in a plate reader. Three biological replicates with six technical replicates each were analyzed.

#### 2.5.3 Microscopic analysis

L929 cells in all culture wells were examined daily during the experiment under a light microscope (Olympus CKX41, Olympus Europa SE & Co. KG, Hamburg, Germany). Microscopic imaging of representative wells was performed using a Cytation 5 Cell Imaging Multi‐Mode Reader (BioTek Instruments GmbH, Bad Friedrichshall, Germany). Imaging was performed in brightfield using the intrinsic auto‐exposure function of the Gen5 imaging software (Version 3.10.06, BioTek Instruments GmbH, Bad Friedrichshall, Germany) for 4x or 20x objectives.

### 2.6 HEK 293E culture conditions and viability assessment

HEK 293E cells (MEXi‐293E cells, IBA Lifesciences GmbH, Göttingen, Germany) were routinely cultivated in 125 mL shake flasks (Thermo Fischer Scientific Inc, Waltham, USA) in MEXi‐CM (IBA Lifesciences GmbH, Göttingen, Germany) supplemented with 8 mM L‐glutamine (Sigma‐Aldrich Chemie GmbH, Steinheim, Germany) and 50 mg x L^‐1^ Geneticindisulfat (G418)‐solution (Carl Roth GmbH, Karlsruhe, Germany) in a 5% CO_2_, humidified atmosphere at 37°C (Heracell vios 160i CO_2_ incubator, Thermo Fisher Scientific Inc., Waltham, USA) at a shaking rate of 190 rpm with an orbital diameter of 19 mm. Experiments were performed with cells of passage number up to 15. At the start of an experiment, 50 mL cultivation tubes (Tubespin Bioreaktor 50, Techno Plastic Products AG, Trasadingen, Switzerland) were filled with the related EM (see Section 2.3) or control medium and inoculated with 0.3 x 10^6^ cells⋅mL^−1^. The starting volume of each culture was 10 mL and the shaking rate was adjusted to 210 rpm. After 24, 48, and 72 hours, a sample of 0.5 mL was taken from each culture, and the viable cell density (VCD) and viability of the culture were analyzed using a trypan blue assay–based Cedex cell counter (Cedex HiRes, Roche Diagnostics GmbH, Mannheim, Germany). Three biological and three technical replicates were analyzed, and mean and standard deviation were calculated for the technical replicates.

### 2.7 Saccharomyces cerevisiae culture conditions and growth studies

*S. cerevisiae* NCYC 1024 cells (National Collection of Yeast Cultures, Norwich, United Kingdom) stored at ‐80°C with 15 % glycerol (Carl Roth GmbH, Karlsruhe, Germany) were resuspended in 10 mL yeast extract peptone dextrose (YPD) medium (constituted of 10 g L^‐1^ yeast extract, 20 g L^‐1^ peptone and 20 g L^−1^ glucose, all Carl Roth GmbH, Karlsruhe, Germany) adjusted to pH 5.8 using 2 M HCl (Carl Roth GmbH, Karlsruhe, Germany) and supplemented with 34 μg mL^−1^ chloramphenicol (Sigma‐Aldrich Chemie GmbH, Steinheim, Germany). The cells were incubated overnight (14 h) at 200 rpm and 30°C in 50 mL cultivation tubes (Greiner Bio‐One GmbH, Germany) using a shaking incubator (IKA^®^ KS 4000 ic control, IKA^®^‐Werke GmbH & Co. KG, Germany). Subsequently, this pre‐culture was used to inoculate the respective extraction and control medium to an optical density at 600 nm (OD_600_) of ∼ 0.2 in a final volume of 12.5 mL in 125 mL shake flasks (Thermo Fisher Scientific Inc., Waltham, USA). These flasks were again incubated at 30°C and 200 rpm; samples were collected during an incubation period of 12 h, and OD_600_ measurements were performed using a spectrophotometer (Libra S50, biochrom Ltd, United Kingdom). The experiment was repeated for three different pre‐cultures with three technical replicates each for the EM and control medium.

## 3 RESULTS AND DISCUSSION

### 3.1 L929 cultivation in extraction medium

**FIGURE 2:**
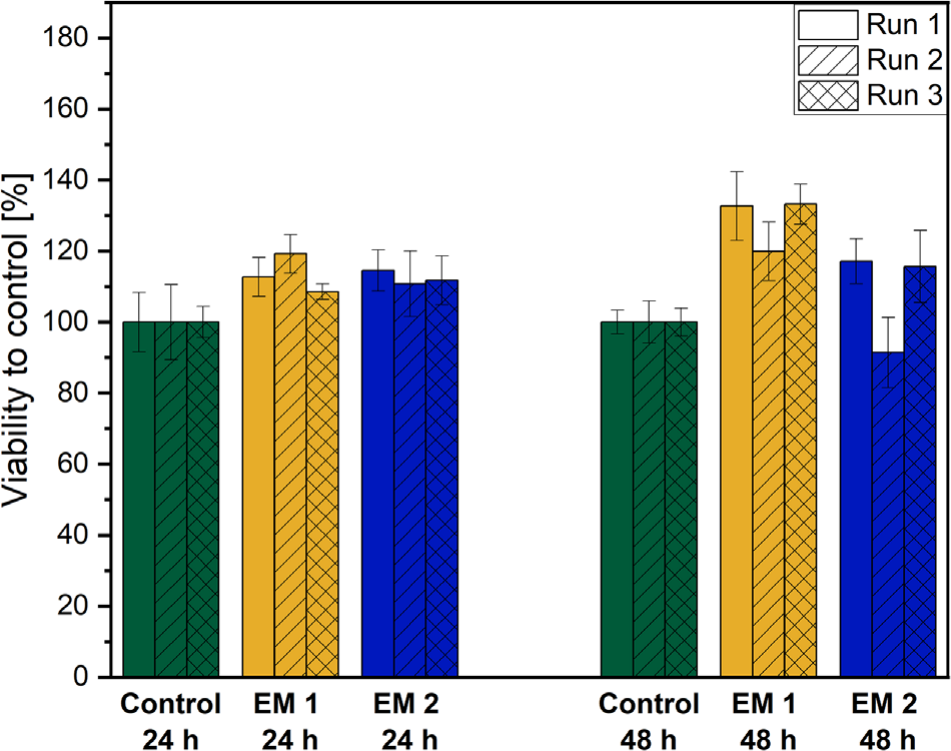
Results of CellTiter‐Blue® cell viability assay to analyze the metabolic capacity (shown as cell viability in %) of L929 cells during cultivation in extraction medium (EM) compared to regular cell culture medium (control). EM 1: EM obtained by incubation of 3D‐printed cubes treated with ethanol (70%, v/v). EM 2: EM obtained by incubation of autoclaved 3D printing material. The experiment was repeated three times, the results of each run are shown as mean ± standard deviation. The cell viability is normalized to the control cultivation that is defined as 100% viability

L929 cells are considered a standard for biocompatibility testing as these cells are recommended by the international organization for standardization and, therefore, are commonly used in laboratories for such purposes [35, 45]. In this work, the potential cytotoxicity of the novel heat‐resistant 3D printing material to L929 cells was assessed using the CTB cell viability assay; this test analyzes the metabolic activity of cells as an indicator of their viability. As shown in Figure 2, the CTB assay reveals that ethanol (70%, v/v) as a disinfectant and autoclaving for sterilization of the 3D printing material did not negatively affect the metabolic capacity of L929 cells, emphasizing the biocompatibility of the 3D printing material for this cell type. In fact, the cells cultivated in both EM for 24 h showed slightly higher mean metabolic activities of 113.5 ± 5.4 % (EM 1) and 112.4 ± 2.0 % (EM 2) compared to the control that is defined as 100% viability. Also, after 48 h of cultivation, the cells’ mean metabolic activity in both EM remains higher compared to the control with 128.6 ± 7.5 % for EM 1 and 108.1 ± 14.4 % for EM 2. However, this increase may not be significant and could be further investigated in future studies.

**FIGURE 3:**
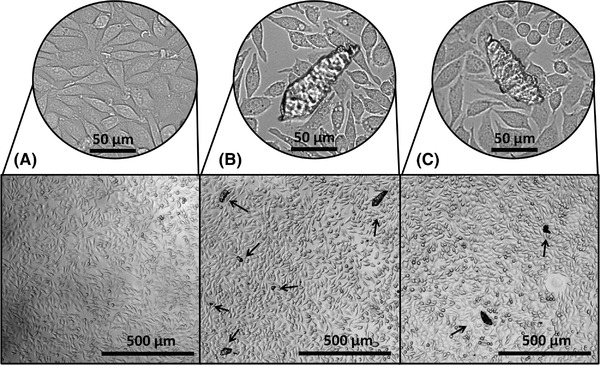
Microscopic images of L929 cells after 24 h cultivation in regular cell culture medium (A), EM 1 (B), and EM 2 (C). Black arrows indicate particles observed in the media. The magnifications show a characteristic spot within the same well of a 96‐well cell culture plate as the corresponding lower magnified pictures.  (EM 1: Extraction medium obtained by incubation of ethanol (70 %, v/v) disinfected 3D‐printed cubes; EM 2: Extraction medium obtained by incubation of autoclaved 3D‐printed cubes)

Microscopic analysis of the L929 cells supports our finding that the material shows biocompatibility with this cell type; as demonstrated in Figure 3, the cells show a similar confluence and an unaltered cell morphology in both EM 1 and EM 2. Yet, the microscopic images also reveal the presence of particles in EM 1 and EM 2 but not in the control medium. Thus, we conclude that the particles stem from the 3D printing material; this is emphasized by their angular shape and translucency, which is consistent for all particles. The formation of particles is probably associated with the layer‐by‐layer fabrication process and the resulting high roughness of the 3D printing material. Despite the presence of the particles, no reduction in cell growth or change in morphology of the directly adjacent cells was observed. Therefore, we envision that L929 cells can also be cultivated inside 3D‐printed cell culture systems in direct contact with the material.

**FIGURE 4:**
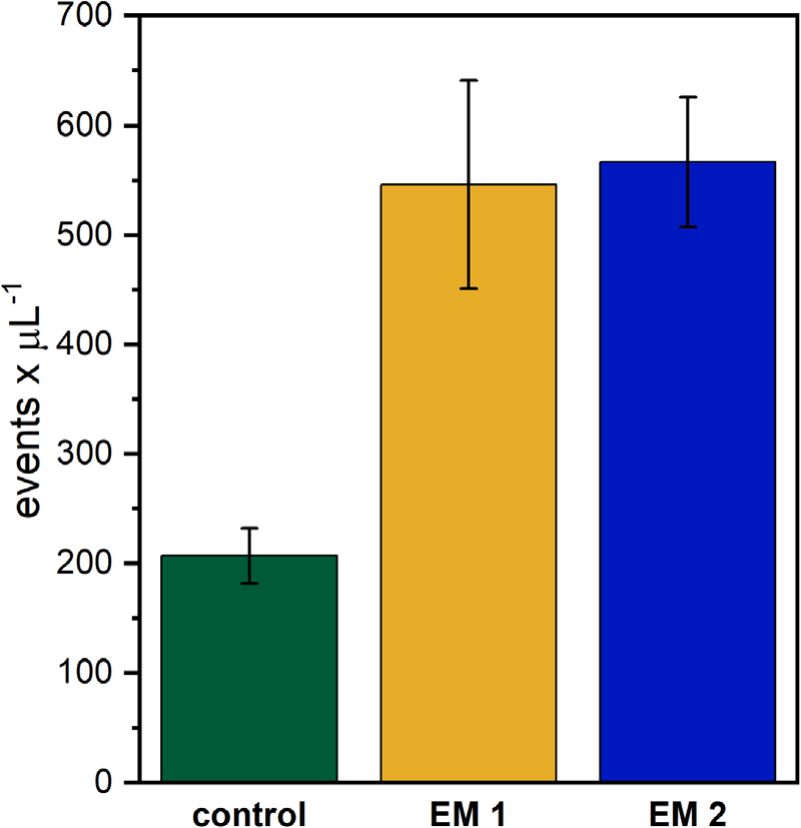
Events detected by flow cytometry in the regular cell culture medium (control) and the extraction media (EM). (EM 1: Extraction medium obtained by incubation of ethanol (70 %, v/v) disinfected 3D‐printed cubes; EM 2: Extraction medium obtained by incubation of autoclaved 3D‐printed cubes). Three technical replicates of three biological replicates of each medium were analyzed; the results are shown as mean ± standard deviation

The extraction media were analyzed by flow cytometry to assess the size and amount of the observed particles. Each event detected by the instrument corresponds to one particle. As presented in Figure 4, considerably more events were detected in the EM compared to the control with a 2.64‐fold and 2.74‐fold increase for EM 1 and EM 2, respectively. Moreover, the comparison to a size calibration standard showed that the fraction of particles larger than 4 μm was 4.1 % ± 1.4 % for the control, 13.1 % ± 1.45 % for EM 1, and 7.9 % ± 1.2 % for EM 2. Consequently, most of the particles can not be observed under a standard light microscope. The higher number of particles in the EM in combination with their observed angular shape evidently indicates the detachment of material from the 3D‐printed cubes. Since no obvious deformation of the 3D‐printed cubes after any post processing step was observed, their surface was further analyzed microscopically. Resulting from the 3D printing process three distinct surfaces can be differentiated (Figure S1). Especially the rough surfaces of the X and Y planes show potential for the detachment of particles. Indeed, the X surfaces of the cubes analyzed after incubation in medium were missing parts of their characteristic surface patterns (Figure S2). These findings further indicate the 3D printing material as the origin of the particles. Our results emphasize that in the design of biocompatibility studies for 3D printing materials, not only the presence of leachables but also particle formation should be considered.

### 3.2 HEK 293E cultivation in extraction medium

**FIGURE 5:**
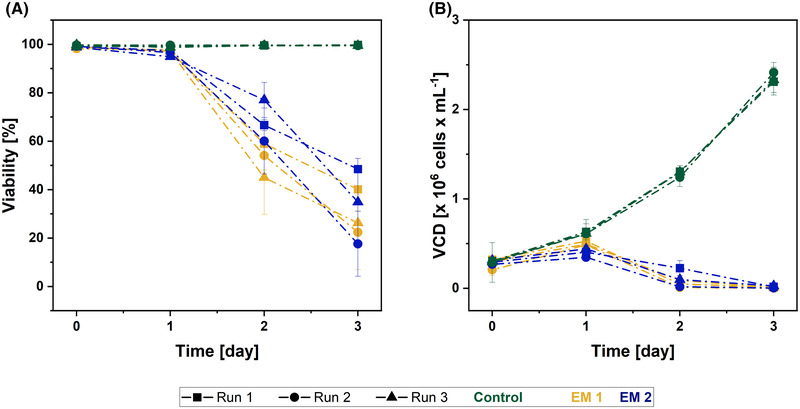
Viability and VCD of the HEK 293E cells during the cultivation in extraction medium (EM 1: obtained by incubation of ethanol (70 %, v/v) disinfected 3D‐printed cubes; EM 2: obtained by incubation of autoclaved 3D‐printed cubes) and regular cell culture medium (control). The experiment was repeated three times, the results of each run are shown as mean ± standard deviation

The mammalian suspension cell line HEK 293 is widely used for academic or pharmaceutical research [46] and, therefore, was chosen as a cell line in our biocompatibility studies. The cell viability and VCD of HEK 293E cells during the cultivation in EM 1, EM 2, and control medium were monitored using a trypan blue assay‐based Cedex cell counter over a period of three days and are presented in Figure 5. The cell viability of HEK 293E cells cultivated in the control medium remained above 99% (see Figure 5A) and a VCD slightly above 2.3 x 10^6^ cells  mL^‐1^ was reached at the end of the experiment (see Figure 5B). In contrast, the cell viability of HEK 293E cells cultivated in both EM decreased considerably from the first day of cultivation. On the second cultivation day, the cell viability decreased below 78 % for all cultures; a trend that proceeded until the end of cultivation. Accordingly, the VCD of these cultures continuously decreased during the experiment and reached values below the inoculation density at the end of the cultivation process. EM obtained from 3D‐printed cubes disinfected with ethanol or sterilized by autoclaving showed a similar and markedly negative influence on the viability and VCD of HEK 293E cells. In consequence, under the given conditions, the material is not suitable for cell culture applications with HEK 293E cells.

The negative effect of the EM on viability and growth of HEK 293E cells may be explained by microparticles that had first been observed in the EM used for cultivation of L929 cells (see Section 3.1) and were also detected by flow cytometry in the EM prepared for the HEK 293E cultivation (data not shown). While the particles had no effect on the viability of adherent fibroblasts, they potentially damage suspension cells physically during cultivation. Considering the unimpaired cell viability of L929 cells, toxicity induced by leachables is unlikely. To further investigate a potential mechanical impairment of the viability of suspension cells by the observed particles, removal of these particles before cultivation could be tested in future works.

### 3.3 *Saccharomyces cerevisiae* cultivation in extraction medium

**FIGURE 6:**
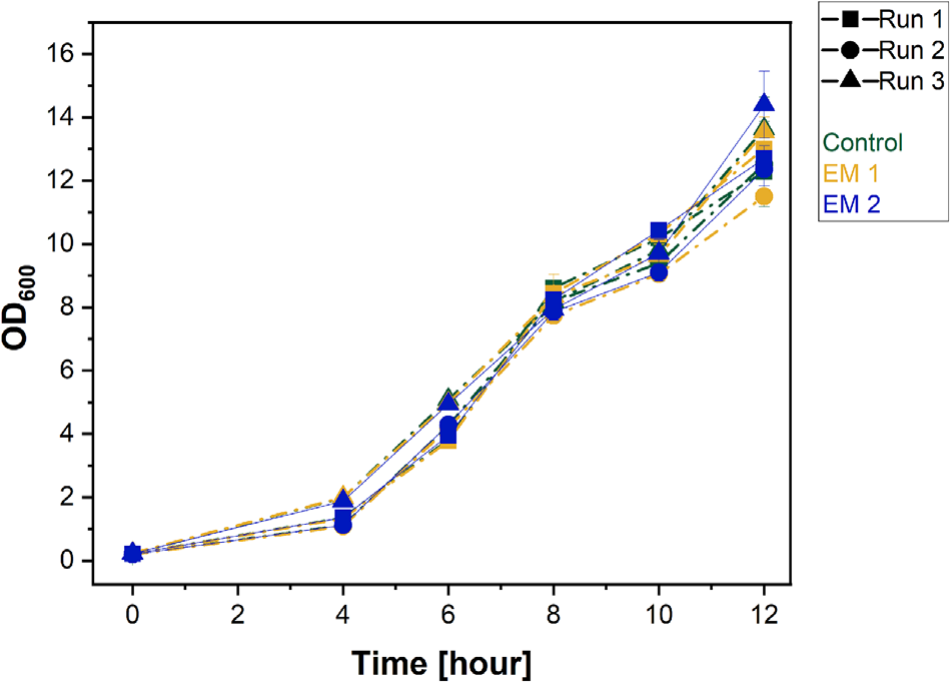
Cell density of *S. cerevisiae* during the cultivation in extraction medium (EM 1: obtained by incubation of ethanol (70 %, v/v) disinfected 3D‐printed cubes; EM 2: obtained by incubation of autoclaved 3D‐printed cubes) and regular cell culture medium (Control) determined by measuring of the optical density at 600 nm. The experiment was repeated three times, the results of each run are shown as mean ± standard deviation

The yeast *S. cerevisiae* is one of the most studied eukaryotes that is frequently used in industrial fermentation processes [47]. Possible applications of 3D printing for yeast cell cultivation can include the design of heat‐steam sterilizable bioreactors that potentially enable flexible adjustments to shifting experimental requirements (i.e., sensor integration). In this study, we investigated the effect of EM 1 and EM 2 compared to the control medium by tracking the OD_600_, a simple but commonly applied parameter for yeast cell culture monitoring (see Figure 6). The growth curves show a typical behavior with a lag phase (0‐4 h), an exponential phase (4‐8 h), a diauxic shift (8‐10 h), and the beginning of a second exponential growth phase (10‐12 h) reaching an OD_600_ of 11‐15 in all cultures after 12 h. For all three individual experiments, the cell growth in both EM remains unaffected; thus, the 3D printing material does not impair yeast cell growth. Furthermore, after about 8 hours of cultivation, the characteristic diauxic shift can be observed as a flattening of the growth curve. At this point, the metabolism switches from glucose as the main energy source to aerobic utilization of ethanol [48]. In comparison to the controls, this critical change in cell metabolism is unaltered in the cultures containing extraction media, underlining the biocompatibility of the material for yeast cell cultivation.

## 4 CONCLUDING REMARKS

In the medical and biotechnology sectors the biocompatibility and sterilizability of 3D‐printed parts is of tremendous importance. In this study, resistance to heat steam sterilization combined with in vitro biocompatibility of a novel heat‐resistant polyacrylate material for high‐resolution 3D Multijet printing was proven with mouse fibroblasts (L929) and yeast cells (*S. cerevisiae*). However, biocompatibility needs to be re‐evaluated for the specific application and cell lines involved—as emphasized by the negative in vitro effect of the material on cell growth and viability of human embryonic kidney cells (HEK 293E) in suspension. This effect may be caused by particles (detached from the 3D printing material) in the extraction media, which may affect the viability and growth of this mammalian suspension cell line. This study therefore highlights the need to consider not only the formation of leachables but also particles in biocompatibility studies, especially for 3D printing materials.

In principle, the investigated 3D printing material shows great potential for rapid prototyping of customized and highly complex cell culture systems due to its resistance to heat steam sterilization, biocompatibility and, capability for high‐resolution 3D printing. This could be of particular interest for the development of new 3D cell culture devices or miniaturized (microfluidic) cell culture platforms.

## CONFLICT OF INTEREST

The authors have declared no conflict of interest.

## Supporting information

Supporting Information

Supporting Information

Supporting Information

## Data Availability

The data that supports the findings of this study is available from the corresponding author upon reasonable request.
