# Supplementary material for: In vitro biocompatibility evaluation of a heat‐resistant 3D printing material for use in customized cell culture devices
Source: Eng Life Sci. 2022 Mar 31;22(11):699–708. doi: 10.1002/elsc.202100104 (PMC9635007; doi:10.1002/elsc.202100104)
Supplement: Supplementary file 1 — Supporting Information [file ELSC-22-699-s002.pdf]

Research Article

In vitro biocompatibility evaluation of a heat-resistant 3D printing material for use in customized cell culture devices

Steffen Winkler[1†], Katharina V. Meyer[1†], Christopher Heuer[1], Carlotta Kortmann [1], Michaela Dehne[1], Janina Bahnemann[1,2]*

Supporting Information

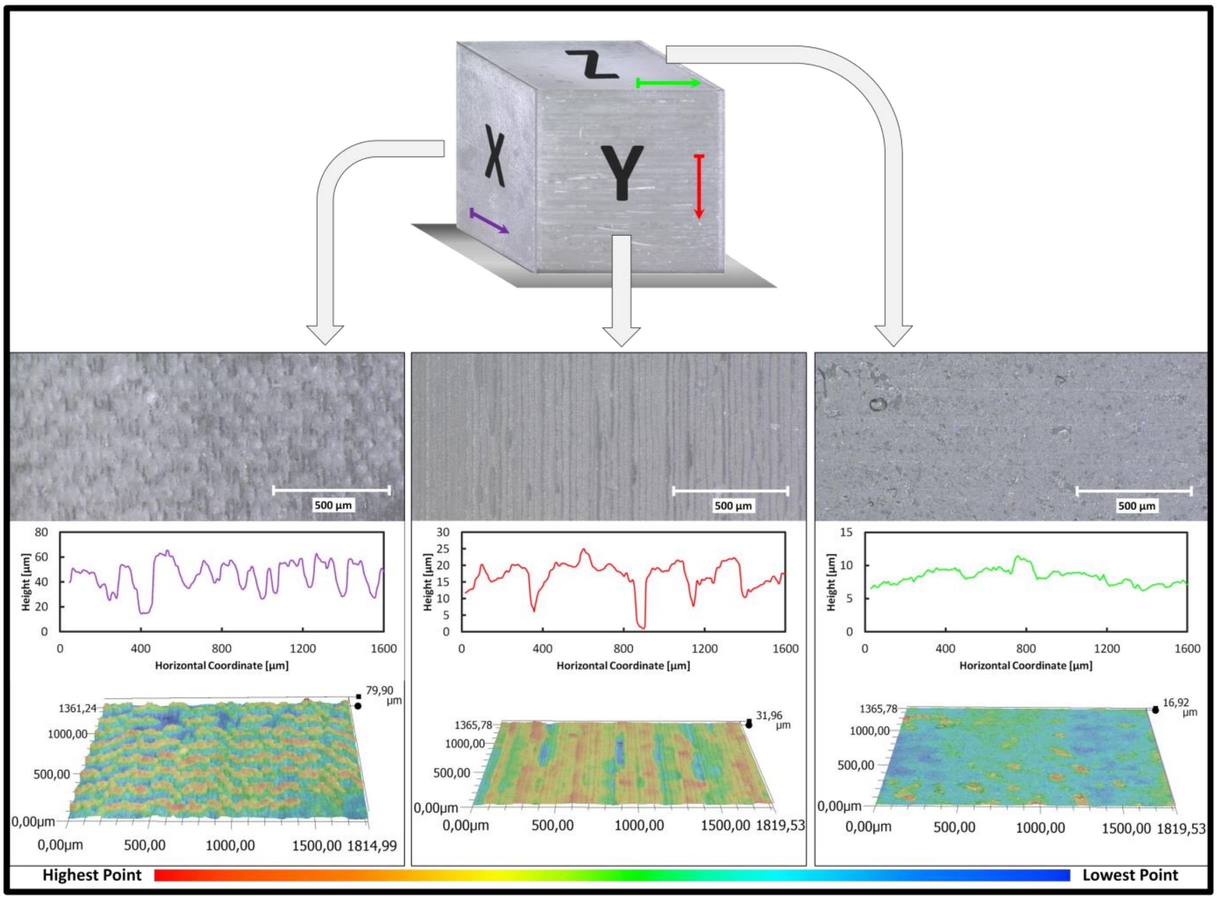

Figure S1: Microscopic analysis of the different surfaces of a 3D-printed cube after post-processing with ddH_2O including height profiles and 3D surface models. Resulting from the 3D printing process three distinct surfaces of the cube can be differentiated. Opposite sides of the cube show a similar surface pattern. The Z plane is defined as parallel to the printing platform and the X plane is defined as being perpendicular to the X axis along which the printing head is moving when creating one material layer. The Y plane is defined as being perpendicular to the X and Z planes. Images were taken in 200x magnification using a VH-Z20UT objective in a VHX-5000 digital microscope (Keyence, Neu-Isenburg, Germany) and the height profile and the 3D surface model were created using its intrinsic Z-stack tool.

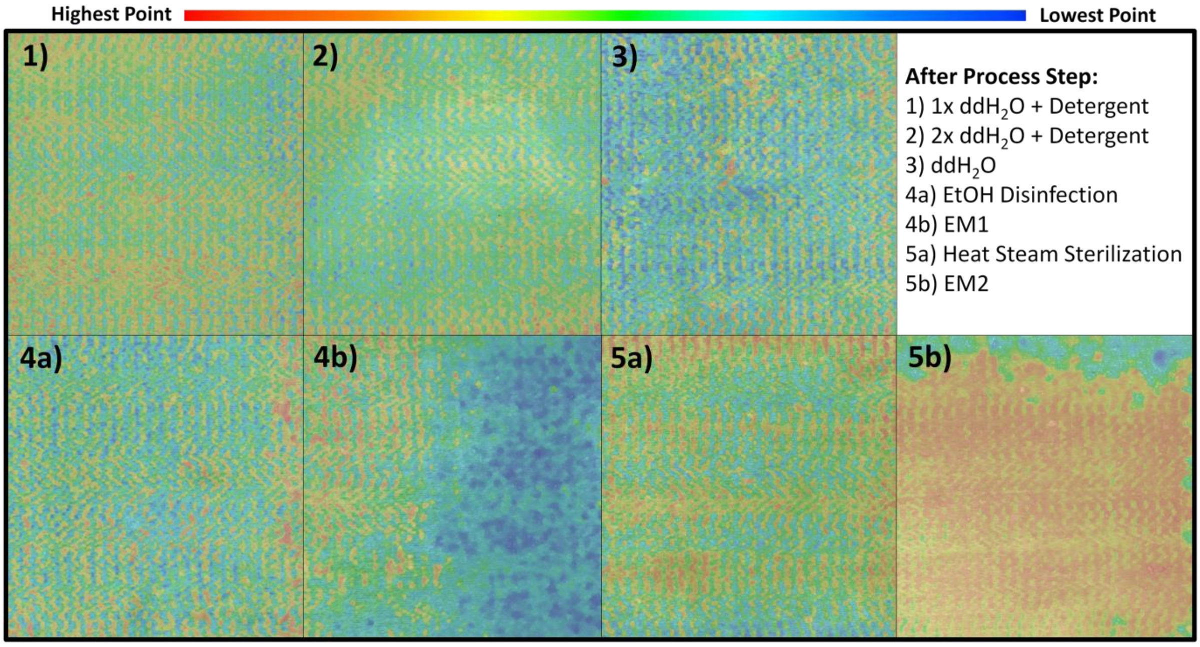

Figure S2: Microscopic images of the X plane (as defined in Figure S1) of 3D-printed cubes after each process step illustrating the surface topography by color. In contrast to cubes obtained after preceding process steps, the surface patterns for cubes obtained from EM1 [4b)] and EM2 [5b)] are incomplete indicating particle detachment. Images with the area of 4 x 4 mm were created by combining the intrinsic Z-stack tool and the montage function of a VHX-5000 digital microscope (Keyence, Neu-Isenburg, Germany) using a VH-Z20UT objective in 200x magnification. A representative image was chosen for each process step.
