# Supplementary figures and images for: In vitro biocompatibility evaluation of a heat‐resistant 3D printing material for use in customized cell culture devices
Source: Eng Life Sci. 2022 Mar 31;22(11):699–708. doi: 10.1002/elsc.202100104 (PMC9635007; doi:10.1002/elsc.202100104)

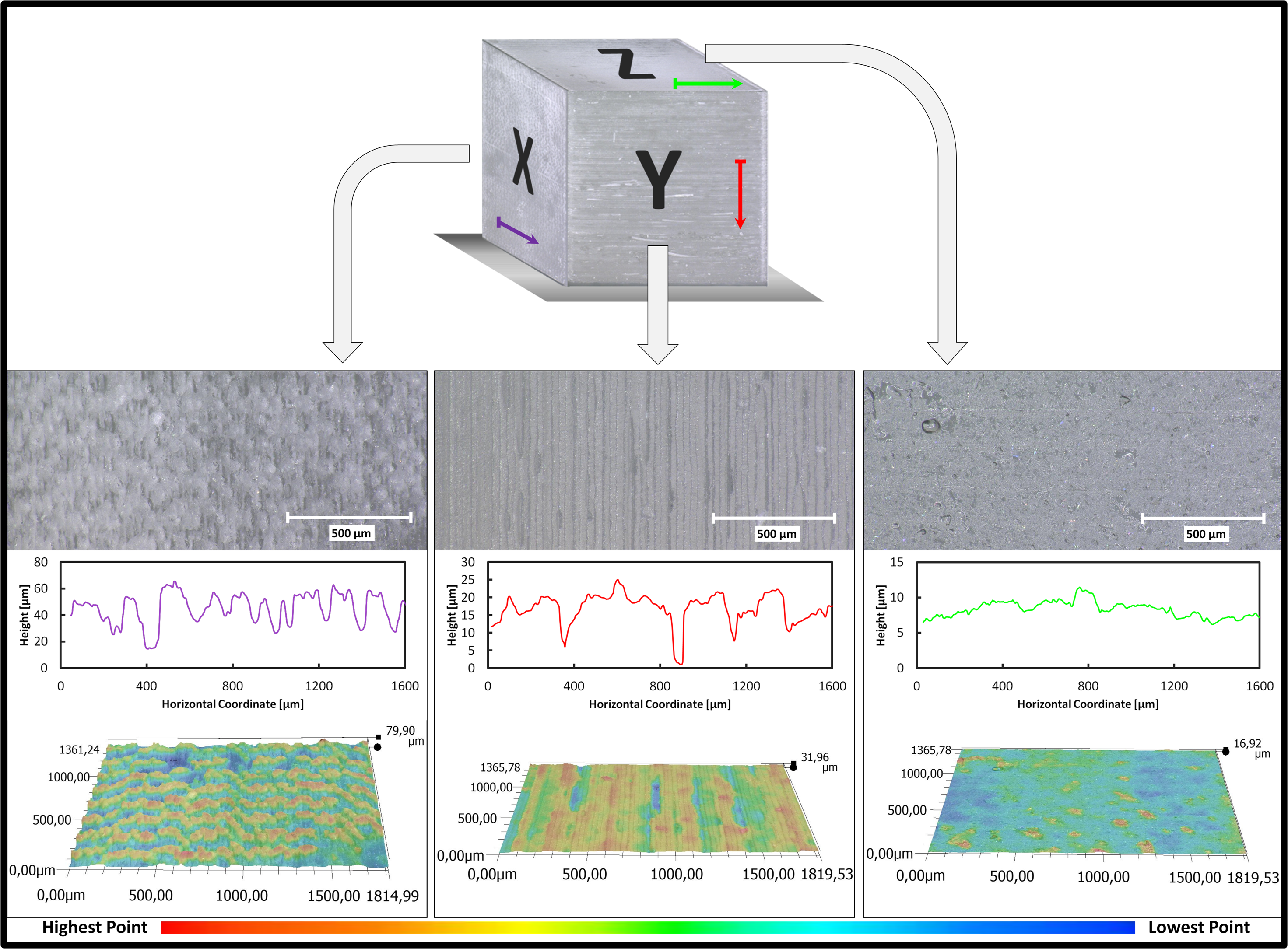

Supplement: Supplementary file 2 — Supporting Information [file ELSC-22-699-s003.png]

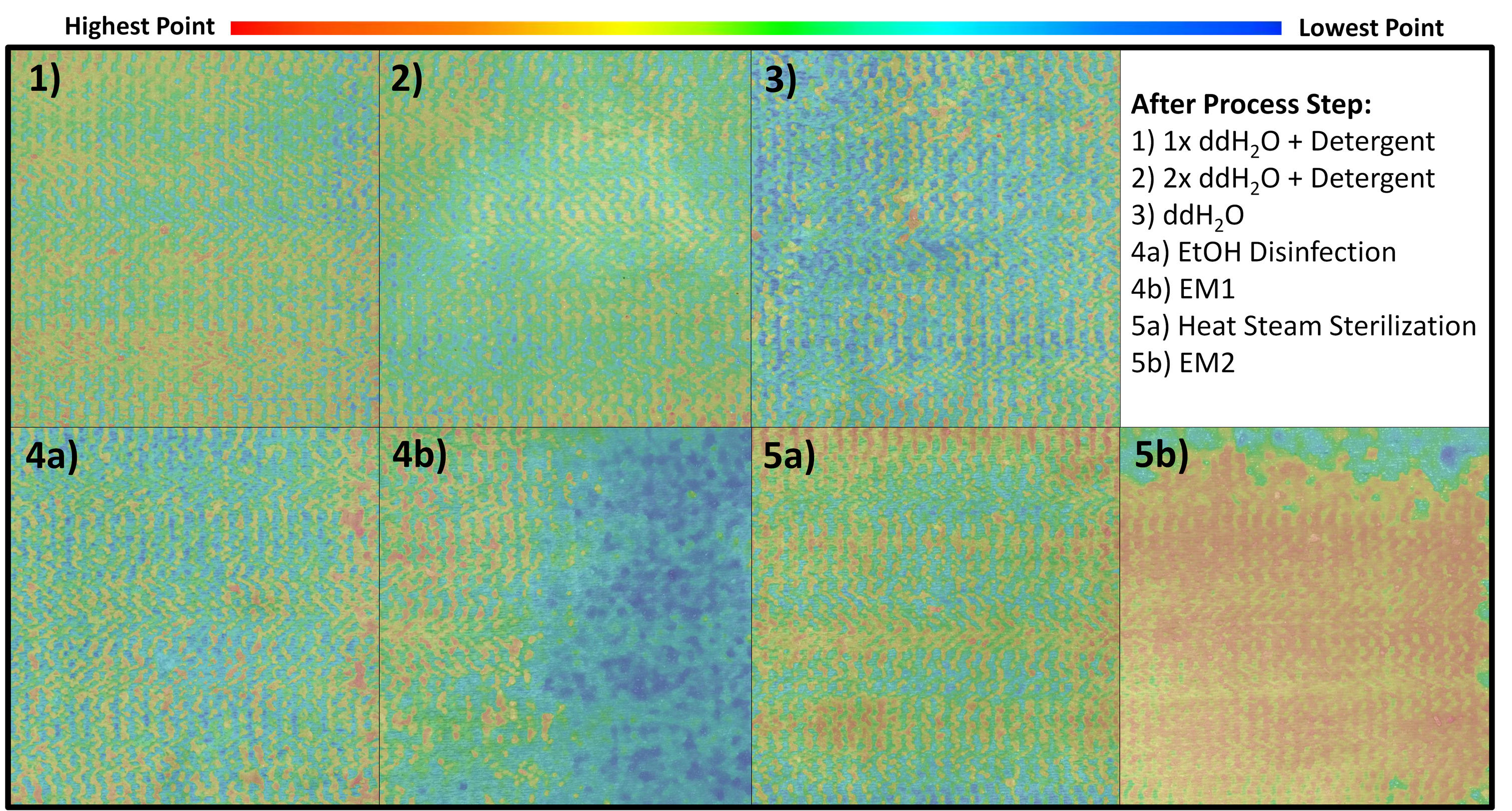

Supplement: Supplementary file 3 — Supporting Information [file ELSC-22-699-s001.png]
